# Epigenetic and microRNA regulation during osteoarthritis development

**DOI:** 10.12688/f1000research.6548.1

**Published:** 2015-10-20

**Authors:** Di Chen, Jie Shen, Tianqian Hui

**Affiliations:** 1 Department of Biochemistry, Rush University Medical Center, Chicago, IL, 60612, USA; 2 Department of Orthopedic Surgery, Washington University, St. Louis, MO, 63110, USA

**Keywords:** Osteoarthritis, microRNA, epigenetics, miRNA, methylation, miR-140

## Abstract

Osteoarthritis (OA) is a common degenerative joint disease, the pathological mechanism of which is currently unknown. Genetic alteration is one of the key contributing factors for OA pathology. Recent evidence suggests that epigenetic and microRNA regulation of critical genes may contribute to OA development. In this article, we review the epigenetic and microRNA regulations of genes related to OA development. Potential therapeutic strategies may be developed on the basis of novel findings.

## Introduction

Osteoarthritis (OA) is the most common form of arthritis and is the leading cause of impaired mobility in the elderly ^1^. It has been projected that more than 67 million people will be affected by OA in the US by 2030, resulting in an extremely high socioeconomic burden ^2, 3^. In recent years, the surgically induced destabilization of the medial meniscus (DMM) model ^4^ and genetic mouse models ^5– 12^ have been developed to delineate the potential roles of affected genes in OA pathogenesis. However, a full understanding of the factors affecting the initiation and progression of the disease has not yet been revealed. Thus, there is no clinical diagnosis for early OA and no effective disease-modifying treatment for late-stage OA, except pain-relieving medication and surgical replacement of the damaged joints ^13– 15^. Compelling evidence has revealed that epigenetic and microRNA (miRNA) alterations occur in OA chondrocytes and in patients with OA, including several well-documented OA-related genes, indicating, to a certain extent, that epigenetic and miRNA regulation contributes to OA pathogenesis ^16– 18^. In this short review, we will summarize the current understanding of OA, speculate on the potential mechanism(s) of epigenetic and miRNA regulation underlying OA development and progression, and in this context propose potential therapeutic targets for the treatment of OA.

## Pathogenesis of osteoarthritis

OA is a degenerative joint disease with major clinical symptoms, including chronic pain, joint instability, stiffness, and radiographic joint space narrowing. During OA progression, articular chondrocytes undergo hypertrophy, leading to extracellular matrix (ECM) degradation and articular cartilage breakdown, followed by vascular invasion, subchondral bone sclerosis, and osteophyte formation eventually developing at the margins of the joint ^19– 21^. OA is a complex multi-factorial disease, and the effects of aging and obesity, mechanical influences, and environmental and genetic factors have been identified as major factors contributing to the initiation or progression (or both) of OA ^22, 23^. Because articular cartilage damage is the primary pathologic feature leading to the joint dysfunction, it has received much of the attention in OA studies. Normal articular cartilage emerges during the postnatal stage as a permanent tissue distinct from the growth plate. The articular cartilage tissue lining the surface of all diarthrodial joints is a smooth, hard, white tissue, which cushions and absorbs the shock between joints. Collagens and proteoglycans are the principal ECM molecules of articular cartilage ^24– 27^. Mutations of ECM-related factors, including, types II *,* IX and XI collagen, have been reported in human OA patients ^28– 30^. It has been established that articular chondrocytes are the cells responsible for maintaining joint cartilage homeostasis. Thus, dysregulation of articular chondrocytes is directly connected to the process of cartilage degradation in OA. An understanding of the phenotypic behavior of articular chondrocytes in homeostasis and disease has revealed several key environmental and genetic factors that impact OA development and progression.

## Genetic contributions to osteoarthritis

A genetic predisposition to OA has been established for many years through several twin studies, segregation analyses, linkage analyses, and candidate gene association studies ^31– 33^. Although the genetics of OA are complex, the genetic contribution to OA is highly significant. It has been demonstrated that the heritability of OA may be as high as 40–65%, depending on the joint site and population studied ^34^. In the past decade, the potential roles of genes and signaling pathways in OA pathogenesis have been demonstrated by *ex vivo* studies with tissue derived from OA patients and *in vivo* studies with surgically induced OA animal models as well as mouse genetic models. Transforming growth factor-beta (TGF-β), Wnt/β-catenin, Indian Hedgehog (Ihh), Notch, fibroblast growth factor (FGF), and hypoxia-inducible factor (HIF) pathways, by stimulating chondrocytes toward hypertrophy, have demonstrated the critical and unique roles of chondrocytes during OA development and progression in genetic mouse models ^5– 7, 9, 10, 35^. These recent genetic findings further suggest that Runt-related transcription factor 2 ( *Runx2*), *Mmp13*, and *Adamts5* are common target genes involved in the above-mentioned signaling networks, disrupting the anabolic and catabolic balance in chondrocytes and eventually degrading the cartilage matrix by upregulation of matrix metalloproteinase (MMP) and a disintegrin and metalloprotease with thrombospondin motif (ADAMTS) activity, which leads to degradation of type II collagen and aggrecan ^8, 11, 36– 38^. Although these studies have been important in determining the genetic components of OA, only a few OA-related genes have been identified by using human genetic and epidemiological approaches. More recent newer technologies, such as genome-wide association studies (GWASs), have been used to analyze large numbers of OA and control populations throughout the world in hopes of uncovering more genes associated with OA. To date, even these larger exploratory human genetic studies have produced very few genes important to the development and pathogenesis of human OA. Whereas some of the genes identified are important structural and ECM-related factors ( *Col2a1*, *Col9a1*, and *Col11a1*) as well as critical signaling molecules in the Wnt ( *Sfrp3*), bone morphogenetic protein (BMP) ( *Gdf5*), and TGF-β ( *Smad3*) signaling pathways, most have been previously implicated in OA or articular cartilage and joint maintenance by using mouse models of induced genetic alteration or surgically induced OA ^28– 30, 39– 42^. New single-nucleotide polymorphisms were identified in several genes, including GNL3, ASTN2, and CHST11, in recent genome-wide screen studies ^43^, and these findings need to be further confirmed.

## Epigenetic alterations in osteoarthritis pathogenesis

In addition to GWAS analyses, growing evidence suggests that the gene expression profile can be largely regulated by epigenetic machinery that modulates local transcriptional activity and mRNA expression in chondrocytes ^44^. In normal adult chondrocytes, like other somatic cells, the genomic arrangement and packaging are regulated by genetic and epigenetic mechanisms that provide instruction on how, where, and when genetic information should be used. In mammals, the major epigenetic regulatory mechanisms include DNA methylation and histone modification. miRNAs could be loosely defined as epigenetic factors and play important roles in OA ^45^.

### DNA methylation

DNA methylation is mediated by DNA methyltransferase (DNMT), which transfers the methyl group from the donor, methylated S-adenosyl-methionine (methyl-SAM), to DNA bases, particularly cytosine (CpG island). DNA methylation occurs in both the gene promoter region and gene bodies and regulates gene transcription ^46– 48^. Recent studies found that DNA methylation is dynamically regulated through a cyclic enzymatic cascade composed of cytosine methylation by DNMTs and demethylation by ten-eleven translocation methylcytosine-(TET) dioxygenases (TET1, 2, and 3) ^49^. In mammals, there are three enzymatically active DNMTs, DNMT1, DNMT3a, and DNMT3b, and one related regulatory protein, DNMT3L ^48^. DNMT1 is primarily a “maintenance” methyltransferase that recognizes the hemi-methylated DNA strand and preserves the methylation pattern throughout cell replication and division. The global knockout of the *Dnmt1* gene is embryonically lethal at E10.5 because of a significant loss of global DNA methylation, suggesting that DNA methylation is essential for normal mammalian development ^50^. In contrast, two *de novo* DNMTs, 3a and 3b, have tissue-specific expression patterns and create unique methylation signatures. Knockout mice with *Dnmt3b* deletion showed embryonic lethality between E11.5 and E15.5 as well as several skeletal defects, including growth impairment. However, loss of the *Dnmt3b* gene does not affect the entire genome methylation pattern ^51^.

In recent decades, researchers have studied changes in the DNA methylation status of individual genes during OA development and progression and found that the promoter of *Col10a1* appeared to be hypomethylated during chondrocyte hypertrophy and maturation followed by its upregulation ^52^. Similarly, the CpG sites within the promoter area of a number of metalloproteinases, including MMP2, MMP9, MMP13, and ADAMTS4, showed decreased methylation profiles in OA compared to normal cartilage, correlating with elevated gene expression and resulting in ECM degradation ^53, 54^. Reduced CpG methylation was reported in the *MMP13*, *IL-1β*, and inducible nitric oxide synthase ( *iNOS*) promoter in OA tissue which correlates with the increased *MMP13*, *IL-1β*, and *iNOS* expression in OA chondrocytes ^55, 56^. During the chondrocyte maturation process, changes in DNA methylation patterns were observed in several transcription factors, such as Sox9 and Runx2 ^57^. Hypomethylation in promoter regions of those genes promoted gene transcription, which further activated downstream signaling molecules, including MMPs, and eventually stimulated chondrocytes toward hypertrophy and terminal maturation. Either hypomethylation or hypermethylation occurred in promoter regions within a subset of OA-specific genes, including ligands (e.g., BMP7 and IL-1β) ^58, 59^, receptors, transcription factors (e.g., Sox9 and Runx2) ^57^, enzymes (e.g., MMPs and ADAMTS4/5) ^53, 54^, and ECM proteins (e.g., aggrecan, Col2a1, and Col10a1) ^52, 60^.

Recent methylome screening data further confirmed that alterations in DNA methylation occurred in OA chondrocytes and that chondrocyte transcriptomes may be changed in OA patients, indicating that DNMTs influence OA susceptibility and severity by modulating pathways or signals leading to OA ^16– 18, 61, 62^. However, which DNMT factor or factors mediate these changes genome-wide remains largely unknown. In one of our ongoing experiments, we have found that DNMT3b, but not DNMT 1 or 3a, was highly expressed in articular chondrocytes, but its expression was significantly decreased in chondrocytes derived from patients with OA or from several OA mouse models, including the aging animal model, meniscal ligamentous injury (MLI) model, and obesity model (Shen *et al.*, unpublished data). Recent reports demonstrated that TET1, 2, and 3 are present in human chondrocytes and that *TET1* expression was significantly reduced by inflammatory factors, such as IL-1β or TNFα ^63^. Recent studies have also revealed a significant increase in 5-hydroxymethylcytosine levels in OA chondrocytes because of TET1 downregulation ^64, 65^. Because DNA methylation is a reversible process, the role of the TET family members in OA development needs further investigation to better understand the regulation of DNA demethylation during OA development and progression.

The regulation of transcription factors on chondrocyte-specific genes through alterations of DNA methylation and histone modification has been reported in recent years. For example, it has been reported that methylation of the -110 bp CpG site in the *Mmp13* promoter strongly correlates with the high *Mmp13* expression in chondrocytes. This CpG site resides within a HIF consensus motif. The methylation of this site will decrease HIF-2α binding to the *Mmp13* promoter ^55^. AT-rich interactive domain 5b (Arid5b) is a newly identified transcriptional co-regulator of Sox9. Arid5b recruits Phf2, a histone lysine demethylase, to the promoter region of Sox9 target genes and stimulates H3K9me2 demethylation of these genes. In the promoters of chondrocyte marker genes, H3K9me2 levels are increased in Arid5b knockout chondrocytes ^66^.

### Histone modification

Working closely with DNA methylation, histone modification—including acetylation, phosphorylation, methylation, and ubiquitination—regulates gene expression by controlling the accessibility of the transcriptional machinery ^67, 68^. Recent studies demonstrated that histone acetylation and deacetylation are involved in OA pathogenesis by affecting chondrocyte anabolic and catabolic processes. Histone acetylation is mediated by histone acetyltransferases (HATs) and is a critical step in loosening the DNA structure, which allows regulatory factors to access the transcriptional machinery and the subsequent initiation of gene expression, whereas deacetylation is considered the termination or repression of gene expression ^69^. Histone deacetylation is mediated by histone deacetylases (HDACs), including the classic HDAC and NAD ^+^-dependent silent information regulator 2 (SIR2) families ^70, 71^. The use of large-scale analysis (ChIP-seq) of chondrocyte histone acetylation did not find global alterations in OA chondrocytes but did find changes in specific gene loci, encoding MMPs, ECM molecules, and inflammatory factors.

In patients with OA, elevated HDAC7 expression has been reported to contribute to cartilage degradation by inducing *Mmp13* expression in OA cartilage. The inhibition of HDAC7 *in vitro* leads to suppression of inflammatory factor-induced *Mmp13* expression ^72^. The expressions of HDAC1 and HDAC2 are upregulated in OA synovial tissue as well, and this may lead to repression of *Col2a1* expression in chondrocytes by interfering with the recruitment of Snail ^73, 74^. Therefore, HDAC inhibitors have been extensively studied in various OA models. Specific HDAC inhibitors can inhibit cytokine-induced MMP expression in chondrocytes to protect against proteoglycan loss and cartilage degradation ^75– 77^. HDAC inhibitors can also stimulate the expression of ECM components—such as Col2a1, cartilage oligomeric matrix protein (COMP), and aggrecan—in chondrocytes ^74, 78^. In the rabbit anterior cruciate ligament transection (ACLT) model, an HDAC inhibitor significantly decelerated injury-induced cartilage erosion, mainly due to reduced expression of MMPs and inflammatory cytokines, indicating that HDAC inhibitors may provide a potential treatment for OA ^79^.

In the SIR2 family, SIRT1 has been extensively studied. SIRT1 is highly expressed in chondrocytes and its expression was found to be decreased in OA cartilage ^80, 81^. SIRT1 can promote expression of ECM genes, such as *Col2a1*, *Col9a1*, and *COMP*, possibly through deacetylation of Sox9, while inhibiting *Col10a1* and *Adamts5* ^82^. SIRT1 also prevents apoptosis in chondrocytes by enhancing insulin-like growth factor (IGF) signaling to inactivate p53. The reduction of SIRT1 expression leads to an increase in chondrocyte apoptosis in OA cartilage ^83^. Interestingly, the function of SIRT1 is closely linked to the inflammatory response and the hypoxic response as well, although SIRT1 has not been approved for use to treat OA. In a variety of tissues, SIRT1 initiates a gene-specific transcriptional repression program to terminate inflammatory response by deacetylating the p65 subunit of nuclear factor-kappa-B (NF-κB) and blocking NF-κB binding to the DNA elements ^84, 85^. SIRT1 can also directly deacetylate and activate HIF-2α, which is upregulated in OA cartilage, to promote MMP expression and eventually degrade the articular cartilage ^86, 87^.

In addition to histone acetylation, histone H3K4 methylation mediated by histone-lysine N-methyltransferase (HMT) was recently investigated. HMT expression level was found elevated in OA cartilage, which resulted in H3K4 methylation at the iNOS and COX-2 promoter areas and induction of gene expression ^88^. Similarly, an age-dependent increase in H3K4me2 occurs in the nuclear factor of activated T cells 1 ( *Nfat1*) promoter, which led to suppression of *Nfat1* expression in adult articular chondrocytes and eventually developed OA-like phenotype in mice ^89, 90^. Increased demethylation mediated by histone demethylase LSD1 was also found in OA chondrocytes. Elevated LSD1 contributed to H3K9 demethylation in the microsomal prostaglandin E synthase 1 (mPGES-1) promoter and induction of gene expression in human OA chondrocyte ^91^. Moreover, the architecture of histone acetylation and methylation in local genome can further guide the long-range chromatin interaction to regulate specific gene regulatory DNA elements ^92^.

## MicroRNA regulation

### The role of miR-140 in osteoarthritis pathogenesis

miRNAs are endogenous non-coding RNAs and play important roles in negative regulation of RNA stability and protein expression ^93, 94^. Several miRNAs have been found to be more abundant in articular chondrocytes than in undifferentiated mesenchymal stem cells. The best example of this is miR-140 ^95^. miR-140 is found in an intron of the *Wwp2* gene coding for WWP2 E3 ubiquitin ligase ^96^. Deletion of *miR-140* did not alter the expression level of *Wwp2* in chondrocytes ^97^. Analysis of the intronic sequence found two miR-140s: miR-140-5p and miR-140-3p ^98^. The expression levels of miR-140-5p and -3p were both significantly reduced in OA chondrocytes ^98^. During chondrocyte differentiation, *miR-140* expression increased in parallel with *Sox9* and *Col2a1*. However, in OA tissues, *miR-140* expression is reduced and *Adamts5* expression was upregulated ^95^. *In vitro* treatment of chondrocytes with IL-1β suppresses *miR-140* expression ^95^. miR-140 is the only miRNA with a cartilage-specific expression pattern ^95, 99^. *miR-140* deficiency accelerates chondrocyte differentiation into hypertrophic chondrocytes and inhibits differentiation of resting chondrocytes into columnar proliferating chondrocytes ^100^. The reduction in *miR-140* expression in OA cartilage may contribute to abnormal gene expression during OA development ^95^. For example, miR-140 regulates the expression of histone deacetylase 4 (HDAC4), a co-repressor of *Runx2* and myocyte-specific enhancer factor 2 ( *Mef2*) ^101^. miR-140 also targets *Cxcl12* ^99^ and *Smad3* ^102^, both of which are implicated in chondrocyte differentiation. In *miR-140* null mice, OA-like changes were observed and characterized by proteoglycan loss and fibrillation of articular cartilage, probably due to increased *Adamts5* expression ^103^. This increased *Adamts5* expression was reversed by transfection of ds-miR-140 into *miR-140*-deficient chondrocytes ^103^. In addition, cartilage-specific *miR-140*-overexpressing transgenic mice had no abnormal skeletal phenotype during embryonic development but did show a protective effect in an antigen-induced arthritis model ^103^. However, the upregulation of *Adamts5* and *Hdac4* expression in chondrocytes was not found in the other *miR-140* knockout mouse model generated by Nakamura *et al.* ^97^. Instead of upregulation of *Hdac4* expression, miR-140 enhances HDAC4 function in chondrocytes ^100^. miR-140 could interact with PTHrP-HDAC4 pathway to control chondrocyte differentiation. *miR-140* deficiency and *PTHrP* or *Hdac4* heterozygosity synergistically impair skeletal growth. Loss of *miR-140* upregulates MEF2C expression. miR-140 negatively regulates p38 mitogen-activated protein kinase (MAPK) signaling, and inhibition of p38 MAPK signaling reduces MEF2C expression ^104^. The functional role of miR-140 in cartilage homeostasis is also involved in the regulation of MMP13 ^105^. MMP13 is a well-known key player in cartilage biology and OA pathology. It has been reported that miR-140 is a negative feedback regulator of MMP13 ^106^. In addition, transfection with pre-miR-140 significantly decreased IGFBP-5 expression. In contrast, transfection with anti-miR-140 significantly increased IGFBP-5 expression ^107^.

### The role of Runx2 in osteoarthritis development

Significant progress has been made in recent years in OA research, and several OA mouse models, including genetic models and surgically induced OA models, have been developed and reported. One common feature of these animal models is upregulation of Runx2 ^5, 9, 36^, leading to further increases in genes coding for matrix degradation enzymes, such as *Mmp9*, *Mmp13*, and *Adamts5*, because Runx2 is a key transcription factor regulating the transcription of these genes ^108– 110^. Key questions are how Runx2 is regulated and whether a therapeutic strategy can be developed by downregulation of Runx2 in OA cartilage.

During skeletal development, Runx2 mRNA expression was detected in skeletal elements as early as E10.5 and E11.5; however, hypertrophic chondrocytes and primary ossification centers do not form until E14.5, although Runx2 is a key transcription factor driving chondrocyte hypertrophy ^111^. These findings suggest that Runx2 protein expression is suppressed because of post-transcriptional regulation during early skeletal development since chondrocyte proliferation and expansion are needed at this stage. These findings also suggest that there is an endogenous negative regulatory mechanism for Runx2 protein expression.

### MicroRNA regulation of Runx2 expression

In recent studies, we have examined potential miRNAs that may bind the 3′-non-coding region of the Runx2 gene and found that miR-204 and miR-211, two homologous miRNAs, bind Runx2 and regulate Runx2 expression in mesenchymal progenitor cells ^112^. To further investigate the functions of these miRNAs in the regulation of Runx2 protein expression in articular chondrocytes and in cartilage homeostasis, chondrocyte-specific miR-204 and miR-211 transgenic mice and conditional knockout mice need to be generated and tested. In addition to miR-204 and miR-211, several other miRNAs have been reported to regulate Runx2 expression ^113^. Their functions in OA development also need further investigation.

The role of miRNA regulation in OA development involves upstream regulation and downstream gene targeting. For example, it has been reported that IL-1β, an inflammatory cytokine, suppresses the expression of miR-140, which in turn causes upregulation of *Adamts5*, a target gene of miR-140, in chondrocytes ^95, 103^, so miR-140 could serve as a mediator during OA development. In addition, it has been reported that TGF-β/Smad3 regulates miR-140 expression in OA chondrocytes ^114^. TGF-β signaling is one of the key signaling pathways in OA development and responds to mechanical loading. Monocyte chemoattractant protein-induced protein 1 (MCPIP-1) is a novel post-transcriptional regulator of IL-6 expression and is targeted by miR-9. MCPIP-1 mRNA expression was low, but expression of miR-9 and IL-6 was high, in damaged OA cartilage. MCPIP-1 protein directly binds with IL-6 mRNA, and overexpression of wild-type MCPIP-1 destabilized the IL-6 mRNA. MCPIP-1 expression was altered by overexpression or inhibition of miR-9. These findings implicate miR-9-mediated suppression of MCPIP-1 in the pathogenesis of OA via upregulation of IL-6 expression in IL-1β-stimulated human OA chondrocytes ^115^. These studies also suggest that miRNAs may serve as important mediators in OA, although they may not be able to trigger the OA occurrence.

## Summary

Although OA is a multi-factorial disease, genetic factors may play a significant role in OA development and progression. Recent evidence suggests that epigenetic and miRNA regulation of genes related to OA development may contribute to OA pathology. To fully understand how mechanical instability and inflammation cause epigenetic and miRNA alteration, further leading to OA development and progression, more in-depth studies need to be conducted. These studies may lead to uncovering novel molecular targets for drug development to prevent and treat OA.

## Abbreviations

ADAMTS, a disintegrin and metalloproteinase with thrombospondin motifs; Arid5b, AT-rich interactive domain 5b; BMP, bone morphogenetic protein; COMP, cartilage oligomeric matrix protein; DNMT, DNA (cytosine-5)-methyltransferase; ECM, extracellular matrix; GWAS, genome-wide association study; HDAC, histone deacetylase; HIF, hypoxia-inducible factor; HMT, histone-lysine N-methyltransferase; IL, interleukin; IGF, insulin-like growth factor; iNOS, inducible nitric oxide synthase; MAPK, mitogen-activated protein kinase; MCPIP-1, monocyte chemoattractant protein-induced protein 1; Mef2, myocyte-specific enhancer factor 2; miRNA, microRNA; MMP, matrix metalloproteinase; Nfat1, nuclear factor of activated T cells 1; NF-κB, nuclear factor-kappa-B; OA, osteoarthritis; Runx2, Runt-related transcription factor 2; SIR2, silent information regulator 2; TET, ten-eleven translocation methylcytosine dioxygenase; TGF-β, transforming growth factor-beta.
